# Retraction Note: Long noncoding RNA MLK7-AS1 promotes ovarian cancer cells progression by modulating miR-375/YAP1 axis

**DOI:** 10.1186/s13046-020-01746-0

**Published:** 2020-11-05

**Authors:** Huan Yan, Hong Li, Pengyun Li, Xia Li, Jianjian Lin, Linlin Zhu, Maria A. Silva, Xiaofang Wang, Ping Wang, Zhan Zhang

**Affiliations:** 1 Department of Obstetrics and Gynecology, The Third Affiliated Hospital of Zhengzhou University, Zhengzhou, Henan People’s Republic of China; 2 Department of Pathology and Laboratory Medicine, University of Tennessee Health Science Center, Memphis, TN USA; 3 Department of Clinical Laboratory, The Third Affiliated Hospital of Zhengzhou University, No. 7 Front Kangfu Street, Zhengzhou, 450052 Henan People’s Republic of China; 4 Department of Bioscience, University of Tennessee Health Science Center, Memphis, TN USA; 5 Collaborative Innovation Center of Molecular Diagnosis and Laboratory Medicine, Xinxiang Medical University, Xinxiang, Henan People’s Republic of China; 6 Department of Reproductive Medicine Center, The People’s Hospital of Henan Province, Zhengzhou, Henan People’s Republic of China

**Retraction Note: J Exp Clin Cancer Res 37, 237 (2018)**

**https://doi.org/10.1186/s13046-018-0910-4**

This article [1] was retracted at the request of the corresponding author. After its publication it transpired that incorrect images have been used in Figure 8A and 8B. The authors have been unable to replace these images. The conclusions of the paper are therefore unreliable.
